# When a Parent Is Born: An Integrated Approach to Perinatal Mental Health and Early Risk Screening

**DOI:** 10.3390/ejihpe15100193

**Published:** 2025-09-25

**Authors:** Claudia Guarneri, Jada Sottile, Eleonora Bevacqua, Maria Clara Leone, Raffaella Mineo, Claudia Rini, Martina Riolo, Antonio Maiorana, Maria Rita Infurna

**Affiliations:** 1 Department of Psychology, Educational Science and Human Movement, University of Palermo, 90128 Palermo, Italy; claudia.guarneri01@unipa.it (C.G.); jada.sottile@unipa.it (J.S.); eleonora.bevacqua@unipa.it (E.B.); raffaella.mineo01@unipa.it (R.M.); claudia.rini@unipa.it (C.R.); martina.riolo@unipa.it (M.R.); 2 HCU Obstetrics and Gynecology, ARNAS Civico Di Cristina-Benfratelli Hospital, 90127 Palermo, Italy; mariaclara.leone@arnascivico.it (M.C.L.); antonio.maiorana@arnascivico.it (A.M.)

**Keywords:** pregnancy, depression, psychological distress, screening, early intervention

## Abstract

This article presents the “When a Parent is Born” project, focused on early identification and intervention for psychological distress during pregnancy and postpartum. It addresses the perinatal vulnerability to depression, and psychological distress, providing psychological support for high-risk cases within a clinical setting. The sample included 997 pregnant women (Mean_age_ = 32.75; SD = 5.33). The protocol encompassed psychological distress (EPDS, PAMA), social support (MSSS), couples’ relationship (DAS), childhood maltreatment (CTQ-SF), and prenatal attachment (MAAS). Univariate and multivariate linear regression models were employed for the analyses. This study highlighted the prevalence of depressive symptoms in 24.7% of the sample, a non-clinical population, and suicidal risk in 2.4%. All predictors were associated with EPDS and PAMA scores in univariate regressions (*p* < 0.005). In the multivariate model of childhood trauma predictors, emotional abuse and neglect were significant for EPDS (F = 19.584, *p* < 0.001) and PAMA (F = 17.876, *p* < 0.001). In the multivariate regression models, the main significant associations (EPDS; F = 17.708, *p* < 0.001) (PAMA; F = 19.346, *p* < 0.001) remained for DAS (*p* < 0.001) and emotional abuse (EPDS *p* = 0.005; PAMA *p* < 0.001). These findings revealed factors associated with perinatal psychological distress and highlighted the importance of psychological screening during pregnancy to support holistic care through a multidisciplinary team. However, the study presents limitations, including the use of self-report measures, the cross-sectional nature of the data, and the limited generalizability of the findings, as the sample is restricted to Southern Italy.

## 1. Introduction

This article presents the ongoing project “When a parent is born”, which proposes a multidisciplinary approach to promoting well-being during the perinatal period. The project is carried out within the Department of Obstetrics and Gynecology at the Civico Hospital in Palermo and is promoted by the Department of Psychology, Educational Science, and Human Movement of the University of Palermo. Specifically, the project promotes the importance of screening psychological and social risk factors during pregnancy and highlights the value of integrating medical and psychological perspectives for a more comprehensive vision of all dimensions that affect women during pregnancy and postpartum. Pregnancy represents a critical window of vulnerability and opportunity, during which the quality of care and support can significantly influence not only maternal health but also fetal development and the couple’s well-being. The WHO (World Health Organization, 1946) asserts that health is “a state of complete physical, mental, and social well-being and not merely the absence of disease or infirmity”. This holistic definition of health is particularly relevant during pregnancy, as it is a period marked by profound biological, psychological, and social changes. From this perspective, the study—and the broader project—adopts a biopsychosocial model, with particular emphasis on the psychological well-being of pregnant women, and its impact on the child and family system. However, despite the recognition of health as a multidimensional construct, perinatal research has often remained fragmented, with a stronger focus on biomedical risk factors than on psychological or relational ones (Alhusen & Alvarez, 2016; Woody et al., 2017). For instance, while systematic reviews have consistently emphasized the prevalence of perinatal depression and its detrimental consequences (Woody et al., 2017; Al-Abri et al., 2023), relatively few studies have examined how different psychosocial risks interact with protective resources in line with the WHO’s biopsychosocial model.

From a clinical perspective, recent guidelines (World Health Organization, 2016) emphasize the importance of prenatal care, including the promotion of health through screening, diagnosis, and prevention. According to these guidelines, a positive pregnancy experience and a fulfilling transition to motherhood are supported by several key factors: the preservation of physical, psychological, and sociocultural well-being; the maintenance of a healthy pregnancy for both mother and baby, which includes the prevention and management of risks, illnesses, and fatalities; a successful transition to positive labor and delivery; and the development of maternal self-esteem, competence, and autonomy. These ideal conditions, however, are not always achieved.

The perinatal period, which typically begins with pregnancy and extends to one year after childbirth, involves substantial physiological and emotional adaptations. For many women, coping with the complex and demanding nature of this transition may contribute to increased vulnerability to mental health difficulties. Indeed, the perinatal period is recognized as a high-risk period for developing depression, anxiety, and other mental disorders, especially in the presence of specific risk factors (Biaggi et al., 2016). A recent meta-analysis provides an overview of the main biological, psychological, and social risk factors associated with perinatal depression (Yang et al., 2022). It particularly emphasizes the importance of not considering these factors in isolation, as some, such as low educational level, poor economic status, and a history of mental illness, are shown to be intrinsically and closely interrelated.

In the Italian context, approximately one in five women experienced some form of perinatal depression (PND). Literature defines perinatal depression as a mood disorder characterized by the onset of depressive symptoms during pregnancy and/or the postpartum period (Terrone et al., 2023; Wikman et al., 2020). Moreover, results from a meta-analysis reported the prevalence of perinatal depression to be approximately 12% (Woody et al., 2017) with a global incidence ranging from 10–20% (Nisar et al., 2020; Wang et al., 2021). Despite its frequency, perinatal depression is frequently underdiagnosed and undertreated, particularly during pregnancy. For example, a study conducted in the United States found that nearly 60% of women experiencing depressive symptoms do not receive a diagnosis, and 50% of women with a diagnosis do not receive adequate treatment (Ko et al., 2012). Similarly, in Italy, the National Institute of Health (Istituto Superiore di Sanità, ISS) has developed guidelines outlining best practices for the screening, prevention, and treatment of perinatal depression (Istituto Superiore di Sanità, 2024). However, these remain recommendations and are, in most cases, not integrated into routine prenatal care, largely due to a predominant focus on medical and obstetric aspects.

From a research perspective, the focus has traditionally been placed on postpartum disorders. However, antenatal depression has been consistently identified in the literature as one of the strongest predictors of postpartum depression (Qi et al., 2023). This implies that implementing screening procedures and providing timely interventions during pregnancy may significantly reduce the likelihood of depressive symptoms persisting or worsening after childbirth (Al-Abri et al., 2023; McGarry et al., 2009; Sidebottom et al., 2021).

Furthermore, antenatal depressive symptoms have been found to not only predict postpartum depression (PPD) but also be associated with obstetric complications such as preterm birth (Jesse et al., 2003), low birth weight (Rahman et al., 2007), and a higher rate of cesarean sections (Chung et al., 2001).

A literature review (Ko, 2017) determined that the current risk factors for antenatal depressive symptoms primarily involved biological, psychological, and social aspects. Biological risks included genetic factors (Mahon et al., 2009), endocrine dysregulation (Yim et al., 2009), and epigenetic factors (Meltzer-Brody, 2011). Meanwhile, sociodemographic and psychosocial characteristics included low socioeconomic status (low level of education, poverty, low income, unemployment), lack of social and partner support, exposure to stressful life events, and depression and anxiety during pregnancy (Beck, 1996; Vesga-López et al., 2008).

The dimension of social support encompasses the individual’s surrounding network, including family members, friends, and partners. Even after childbirth, a woman’s family and social network continue to be a significant resource. According to several studies, social support is a crucial protective factor against the development of antenatal and postnatal depressive symptoms and psychological distress (Dennis & Letourneau, 2007; Dennis & Ross, 2006). Specifically, women who received strong support from their partners during pregnancy tend to present lower levels of postpartum distress (Stapleton et al., 2012).

In this regard, another important aspect is the level of relational well-being between partners. Some studies have shown that a poor relationship constitutes a psychosocial risk factor for the development of antenatal depressive symptoms. Moreover, women who perceived their partners as supportive tended to experience increased well-being in their roles as women, mothers, and partners (Hopkins et al., 1987; Misri et al., 2000). Relationship satisfaction and dyadic adjustment were key factors for maintaining psychological well-being (Thomas et al., 2017). Consequently, mothers experiencing difficulties in their relationships with their partners were at a higher risk of developing PND (Mangialavori et al., 2019).

Furthermore, the literature indicates that childhood trauma experiences are significant risk factors for the development and persistence of antenatal depressive symptoms and psychological distress (Chapman et al., 2004; Wajid et al., 2020). Childhood trauma encompasses a wide variety of negative early-life experiences that can have lasting psychological, emotional, and physical effects. These traumas include physical, sexual, and emotional abuse and emotional and physical neglect. Some of the consequences of childhood trauma experiences extend beyond an increased risk of suicidality and depressive symptoms during pregnancy (Farber et al., 1996), complications affecting both maternal and infant health postpartum (Möhler et al., 2008). These complications may include adverse birth outcomes like preterm birth, low birth weight (Smith et al., 2016), and the occurrence of physical health problems during pregnancy (Lukasse et al., 2009). Another important dimension that may be related to antenatal depressive symptoms is prenatal attachment (Alhusen, 2008; Misri & Kendrick, 2008). Attachment is the emotional connection and concern that typically develops between the pregnant woman (or parents in general) and the unborn child (Condon & Corkindale, 1997). This bond is regarded as the most fundamental form of human intimacy, representing the initial internalized conception of the fetus, which both parents typically develop and refine throughout pregnancy. The quality of the parent–infant relationship is a crucial factor that significantly influences the child’s subsequent cognitive and emotional development (Pisoni et al., 2014). A review suggested that, despite contrasting opinions present in the literature, there was an association between prenatal attachment and depression, particularly prenatal depression (Rollè et al., 2020). Indeed, depressive symptoms were strongly associated with a sense of detachment from the child (Condon & Corkindale, 1997), but it is not clear from an analysis of the literature which of the aspects determines the other.

In summary, this project aims to investigate aspects, as identified in the literature, that are significantly linked to the onset of psychological distress during the perinatal period, with a focus on prevention and mental health promotion. This project involves the comprehensive screening and management of potentially high-risk situations.

In line with the overarching aims of the project, the present study specifically seeks to examine how the selected variables interact and jointly influence the development of perinatal psychological distress. While previous research has often explored these factors in isolation, such as the impact of childhood trauma on antenatal depression, less is known about how these variables combine and interact to influence psychological outcomes in the perinatal context. By aligning with WHO’s 2016 recommendations on antenatal care, the present study adopts a critical stance: it seeks to identify specific pathways of vulnerability and resilience that may inform preventive interventions and ultimately operationalize the WHO’s holistic vision of maternal health.

The central hypothesis of this study is that specific combinations of risk and protective factors may better predict the presence of perinatal psychological distress. In particular, it is hypothesized that experiences of childhood maltreatment (including abuse and neglect) will be associated with increased vulnerability to perinatal depressive symptoms and psychological difficulties. Conversely, protective factors such as strong perceived social support, secure prenatal attachment, and high-quality couple relationships are expected to buffer against such distress. This integrative approach may contribute to a more nuanced understanding of perinatal mental health, adopting a psychodynamic perspective, and inform the development of targeted preventive interventions.

## 2. Materials and Methods

### 2.1. Project Presentation

The project, “When a Parent is Born—Information, Screening, Prevention, and Early Treatment of Psychological Distress in Pregnancy and Postpartum Depression” (PSN2020 CUP D73C23000010003), conducted in collaboration between the Department of Psychology, Educational Science and Human Movement of the University of Palermo and the Complex Operative Unit (U.O.C.) of Obstetrics and Gynecology at the “Civico—Di Cristina—Benfratelli” Hospital of Palermo, is based on screening for psychosocial risk factors during pregnancy. Data collection occurs at two time points: during pregnancy (T0) and approximately 6 months postpartum (T1). A longitudinal approach was utilized; however, only data from T0, corresponding to the pregnancy period, were presented here. A screening protocol was developed to assess psychosocial dimensions identified in the scientific literature as relevant risk factors for the development of psychopathological outcomes during the perinatal period. This protocol was administered to patients within the hospital’s full-term pregnancy and high-risk pregnancy clinics, in addition to the routine medical assessment. The project targets pregnant women, who are invited to participate in the screening assessment with the active involvement of psychologists and healthcare workers. Data was primarily collected through self-report instruments, using the “Google Forms” platform.

The project aims to identify and intervene early and effectively in high-risk situations, such as the presence of moderate to elevated levels of depressive symptoms, high suicide risk, and the presence of moderate to elevated levels of childhood trauma. The intervention oversees the psychological management of these patients through the support of a psychotherapist within the clinic. This aspect reflects the necessity of integrating figures like psychologists and psychotherapists into a medical equipe to ensure comprehensive care for the pregnant woman’s overall well-being.

### 2.2. Procedure and Ethical Aspects

The data collection was preceded by the completion and signing of informed consent forms, which briefly explained the study’s objectives and specified that individual information and responses provided were anonymous and confidential. The patients were guided and supported by the psychologists in completing the screening questionnaire. The screening protocol comprised various areas: socio-demographic information, pregnancy-related information, psychological distress, perceived social support, quality of the couple’s relationship, childhood trauma experiences, and prenatal attachment. At the end of each week, the data obtained through the screening was analyzed by psychologists to generate a list of patients in high-risk situations, which was then disclosed to medical personnel and the psychotherapist. Subsequently, those patients were contacted by the psychotherapist and followed on their course of labor, delivery, hospital stay, and postpartum.

All procedures in this study were conducted in accordance with the 2024 Declaration of Helsinki. The protocol was approved by the Bioethics Committee at the University of Palermo (167/2023) and the Ethics Committee at the “Civico—Di Cristina—Benfratelli” Hospital of Palermo (142/2022).

### 2.3. Sample

The sample of this study, from the beginning of the project to date, includes 997 pregnant women (Mean_age_ = 32.75; SD = 5.33), predominantly of Italian nationality and residing in the Palermo area. To be eligible, participants met three criteria: were at least 18 years old, were pregnant, and spoke fluent Italian. Most of the pregnant women in the sample (91.0%) were in their third trimester and 70.8% of pregnancies were planned. Twin pregnancies were rare (1.0%) and 5.8% of the participants had used Assisted Reproductive Technology. 27.4% of the pregnancies were considered at high obstetric risk. Previous high obstetric-risk pregnancies were reported by 4.9% of the women, 22.8% had experienced a miscarriage, 0.5% had an intrauterine death, and 5.5% had undergone a voluntary termination of pregnancy.

Sociodemographic characteristics (Table 1) showed that 36.2% of the women had a high school diploma, 27.5% had a Bachelor’s degree, and 20.3% a postgraduate degree or a master’s degree. Regarding occupational status, 51.6% had stable paid work, 21.6% were housewives, and 15.3% were unemployed. Marital status was predominantly cohabiting/married at 89.6%, with 9.6% single and 0.8% separated. 61.4% had a modest standard of living, and 29.7% perceived their status as medium-upper class, owning homes and affording travel.

The sociodemographic profile of our sample closely mirrors national statistics on perinatal mental health, as documented in the ISTISAN 23/16 report (Camoni et al., 2023b). The mean age is 33 years, 61.1% of women report a modest economic condition, 61.4% are in stable paid employment, and the majority are cohabiting or married. This alignment with national data suggests that the study population is broadly representative of the wider context, thereby strengthening the external validity of the findings.

### 2.4. Measures

- A questionnaire on the sociodemographic characteristics of the participants was administered to collect essential information such as the status of the pregnancy and any previous pregnancies, the current trimester, the estimated due date, and details about the current pregnancy to determine if it is single or multiple and if it is a high-risk pregnancy. The information collected also included educational level and the status of the romantic relationship between the parents. Additionally, it included questions on previous miscarriages, perinatal deaths, previous high-risk pregnancies, and voluntary terminations of pregnancy. Finally, it investigated the pre-existence of psychological distress (e.g., anxiety, depression) and inquired if any psychological treatment had ever been undertaken.
- Edinburgh Postnatal Depression Scale (EPDS) (Benvenuti et al., 1999; Cox et al., 1987): EPDS is a self-report questionnaire that assesses psychological distress and emotional state over the past week. It consists of 10 items with responses on a 4-point Likert scale (0–3). Item 10 is particularly significant as it evaluates the risk of suicidal ideation. Higher scores indicate greater distress (possible range 0–30): scores from 0 to 8 are categorized as no risk, 9–11 as medium risk, and 12–30 as high risk. The validated Italian translation by Benvenuti et al. (1999) was used, demonstrating favorable psychometric properties (Cronbach’s Alpha = 0.7894).
- Perinatal Assessment of Maternal Affectivity (PAMA) (Baldoni et al., 2018, 2023): PAMA is a self-report questionnaire used to assess perinatal affective disorders. It consists of 11 items: the first eight items explore specific dimensions of emotional and behavioral challenges using a 4-point scale (0–3); the final three items examine whether the reported experiences are associated with parenthood. The areas covered include anxiety, depression, perceived stress, irritability/anger, relationship problems (including those with partners, family, friends, and work colleagues), abnormal illness behavior (such as somatization, functional medical syndromes, and hypochondriacal complaints), physiological issues (like sleeping, eating, or sexual desire), addictions (including smoking, alcohol consumption, drug use, gambling, and compulsive Internet use), and other risky behaviors. A higher score indicates a greater risk for an affective disorder. The Italian version demonstrates adequate internal consistency (Cronbach’s Alpha = 0.78).
- Maternity Social Support Scale (MSSS) (Dabrassi et al., 2009; Webster et al., 2000): The MSSS is a self-report questionnaire to be administered to both pregnant women and their partners. It allows the assessment of the perceived quantity of care (affection, support, etc.) women receive from their families, partners, and friends. The questionnaire consists of 6 items, and responses are on a 5-point Likert scale ranging from 1 (never) to 5 (very much) to determine how much they felt their significant others’ support. The total score can range from 6 to 30. According to the cut-offs established by the authors who developed the scale (Webster et al., 2000), a score between 6–18 indicates a low level of support; a score between 19–24 indicates an intermediate level of support; and a score above 24 indicates an adequate level of support. This instrument exhibits good psychometric properties.
- Dyadic Adjustment Scale (DAS) (Garbarini et al., 2014; Gentili et al., 2002): The DAS measures relationship satisfaction through a 32-item questionnaire divided into four scales. Scores under 21 typically indicate relational distress. In this study, only the “Dyadic Cohesion” and “Dyadic Satisfaction” scales are used. This instrument is the most commonly used tool for assessing the quality of romantic relationships through responses on a 6-point scale ranging from “All the time” to “Never,” which allows for the evaluation of relational well-being or distress. In the Italian version, the “Dyadic Cohesion” scale shows a Cronbach’s Alpha of 0.67, while the “Dyadic Satisfaction” scale has a Cronbach’s Alpha of 0.82.
- Childhood Trauma Questionnaire—Short Form (CTQ–SF) (Bernstein et al., 2003): The CTQ–SF consists of 28 items designed to assess experiences of childhood trauma, rated on a 5-point Likert scale (1 = never true, 2 = rarely true, 3 = sometimes true, 4 = often true, 5 = very often true). The questionnaire includes five clinical subscales: Physical Abuse, Emotional Abuse, Sexual Abuse, Physical Neglect, and Emotional Neglect. Moderate-severe cutoff scores for each subscale are ≥13 for Emotional Abuse; ≥10 for Physical Abuse; ≥8 for Sexual Abuse; ≥15 for Emotional Neglect; and ≥10 for Physical Neglect. For our study, we used the Italian version of the CTQ–SF, as translated by Sacchi and colleagues (Sacchi et al., 2018). All subscales demonstrate excellent psychometric properties, with a Cronbach’s alpha > 0.87.
- The Maternal Antenatal Attachment Scale (MAAS) (Condon, 1993): MAAS is a questionnaire designed to assess prenatal attachment. The scale consists of 19 items and uses a 5-point Likert scale to evaluate the parent’s feelings towards the child along two dimensions: the quality of attachment and the intensity of concern. Higher scores indicate higher levels of attachment. This instrument exhibits good psychometric properties.

All standardized questionnaires used are summarized in Table 2.

### 2.5. Data Analysis

Descriptive statistics were calculated for all sociodemographic and clinical characteristics, as well as for all psychological dimensions assessed. Linear regressions using the total scores of the instruments (PAMA total score, EPDS total score, MSSS total score, DAS total score, CTQ which includes Emotional Abuse, Physical Abuse, Sexual Abuse, Emotional neglect, Physical neglect, and MAAS total score) were conducted, in order to investigate associations between each independent variables (MSSS, DAS, CTQ, MAAS) and the outcome variables (EPDS and PAMA). Each predictor was initially considered separately in a series of univariate regression models. A subsequent multivariate regression model was then performed, incorporating the CTQ subscales to differentiate among various forms of abuse and neglect in relation to the outcomes. Finally, a more complex multivariate regression model was implemented, in which all independent variables were simultaneously included as a set of predictors of the outcome variables. This model was tested on a subsample of 850 women who reported data on the quality of their couples’ relationship, in order to ensure greater accuracy of the analyses. Consequently, the results will be presented on two separate tables. The selected criterion of statistical significance is *p* < 0.05. Model assumptions were assessed, and no relevant violations were observed.

This study aims to explore the strength and direction of the associations between a set of independent variables—conceptualized as potential risk factors—and two outcome measures: depressive symptoms (EPDS) and psychological distress (PAMA) during pregnancy. By identifying the most significant predictors, this research seeks to highlight key areas of psychological vulnerability, thereby informing the development of targeted prevention strategies and tailored clinical interventions to reduce the risk of PND.

## 3. Results

### 3.1. Descriptive Results

The EPDS showed that 24.7% of pregnant women presented moderate to high levels of depressive symptoms (Table 3).

The PAMA also revealed the potential presence of psychological distress across various domains. In this regard, the presence of anxiety was reported in 38.7% of the sample, making it the most frequently observed condition. Physiological problems, reported by 37.1% of the sample, also represented a notable finding. These results represented our primary variables of interest, as they provide a measure of the psychological distress experienced by the pregnant women in our sample.

The dimension of perceived social support, measured through the MSSS, indicates that most mothers perceived a moderate level of support (32.8%). Furthermore, the dimensions of the Dyadic Adjustment Scale (DAS) revealed a high prevalence of perceived relational well-being (93.2%) in a subsample of 850 women. However, relational distress was still present (5.3% women) and should not be overlooked.

Through an analysis of the different subscales of the CTQ, it was observed that 2.1% of women experienced physical abuse during their childhood, and 1.4% experienced sexual abuse. Of the dimensions related to neglect, emotional neglect appeared to be the most prevalent (3%).

The MAAS revealed that more than half of the pregnant women in the sample (55.7%) presented a moderate level of prenatal attachment. A low level of prenatal attachment was reported by only 4.2% of the women.

### 3.2. Univarate and Multivariate Models

Table 4 presents the results of the univariate and multivariate linear regression models conducted to examine the associations between the proposed predictors and the outcome variables. Specifically, the univariate regressions, each performed separately, revealed that all predictors were significantly and strongly associated with both EPDS and PAMA scores (*p* < 0.005). These associations were positive for childhood trauma dimensions and negative for social support, prenatal attachment, and couple relationship quality.

The multivariate model focusing exclusively on childhood trauma predictors demonstrated that only emotional abuse and emotional neglect remained significant when predicting EPDS (F = 16.156, *p* < 0.001) and PAMA (F = 17.876, *p* < 0.001). In particular, emotional neglect showed strong significance in both models (*p* < 0.001), while emotional abuse was highly significant for PAMA (*p* < 0.001) and marginally substantial for EPDS (*p* = 0.006). All other maltreatment dimensions did not reach statistical significance in the multivariate context.

A further, Table 5 presents a more comprehensive multivariate model, tested on a subsample of 850 pregnant women, including all predictors simultaneously and provides additional insight. In the model predicting EPDS (F = 19.584, *p* < 0.001), the variables that remained significant were prenatal attachment (MAAS; *p* < 0.001), couple relationship quality (DAS; *p* < 0.001), and emotional neglect (*p* < 0.005). The emotional abuse dimension falls at the borderline of statistical significance (*p* = 0.006). Additionally, the model predicting PAMA (F = 19.346, *p* < 0.001) showed significant effects only for emotional abuse (*p* < 0.001) and DAS (*p* < 0.001).

The variables included in both the univariate and multivariate regression models were selected not only based on statistical considerations, but also due to their strong clinical relevance. Consequently, the statistically significant associations identified highlight key areas warranting focused attention and improvement in order to provide effective support for the prevention and management of perinatal psychological distress.

## 4. Discussion

In this study, we examined the dimensions highlighted in the scientific literature as potential risk factors linked to antenatal depressive symptoms and psychological distress during pregnancy.

Our results for the two main variables of interest indicated that a substantial portion of our sample, which was not a clinical sample, exhibited a moderate to high level of psychological distress. Specifically, 24.7% of women presented a moderate to elevated presence of antenatal depressive symptoms, and anxiety symptoms were present in nearly 38.7% of the sample. 24 women (2.4%) presented a suicide risk. Finding elevated levels of antenatal depressive symptoms and psychological distress during pregnancy is clinically concerning, not only because it reflects a compromised state of maternal well-being in the present, but also because antenatal depression has been widely recognized in the literature as a robust predictor of postpartum depression (PPD) (Verreault et al., 2014). The prevalence of PPD is estimated to be around 14% globally (Liu et al., 2022), and around 11% in Italy (Camoni et al., 2023a). Nevertheless, the prevalence rate goes up to 27.5% when the cutoff score > 9 for the EPDS is utilized (Benvenuti et al., 1999).

The transition from antenatal to postnatal depression is well documented, with numerous studies demonstrating that depressive symptoms during pregnancy frequently persist or even worsen in the postpartum period if left unaddressed. A comprehensive literature review by Underwood and colleagues (Underwood et al., 2016) further substantiates this strong association, highlighting the predictive value of antenatal depressive symptoms for the onset of PPD across various populations and contexts. Notably, their review includes findings from longitudinal studies, which emphasize the temporal continuity between antenatal and postnatal depressive conditions and reinforce the importance of early identification and intervention during pregnancy to prevent the escalation of symptoms in the postpartum period. Indeed, raising awareness and implementing targeted prevention efforts helps our team to identify at-risk women early, including those whose psychological suffering may be less visible, yet still clinically significant.

The results of our study confirm the initial hypothesis, indicating that it is the interaction between different psychological dimensions, rather than the effect of individual variables, that is most associated with symptoms of perinatal psychological distress. The exception is the dimension of Social Support, which requires further investigation. This study’s innovative contribution lies in its integration of risk and protective factors within a single analytical model, enabling a more comprehensive understanding of their interplay adopting a psychodynamic perspective. This approach provides a more nuanced perspective on the complexities of perinatal mental health by moving beyond reductionist models and emphasizing the importance of considering dynamic, multifactorial influences.

### 4.1. Association Between Childhood Maltreatment and Perinatal Outcomes

Regarding the role of childhood maltreatment as a risk factor for antenatal depressive symptoms and psychological distress, our data demonstrated that emotional neglect is the most commonly reported form of childhood trauma, followed by physical abuse and emotional abuse. It is crucial to consider due to their long-term impact, also during the perinatal period (Racine et al., 2021). Indeed, literature has shown that exposure to traumatic experiences, particularly those affecting emotional and relational domains within the family context, increase vulnerability to antenatal depressive symptoms (Choi et al., 2022; Choi & Sikkema, 2016; Racine et al., 2020; Tebeka et al., 2021).

Recent studies have demonstrated that women who early experienced emotional neglect presented higher levels of depressive symptoms during pregnancy (Can Caglayan et al., 2023; Infurna et al., 2024; Li et al., 2017) and immediately postpartum (Li et al., 2017; Tebeka et al., 2021). This may be due to the fact that pregnancy represents a unique psychological moment in which a woman is simultaneously confronted with the memory of being a child and the emerging responsibility of caring for one. For those who lack adequate caregiving experiences, this dual perspective can activate unresolved emotional wounds and internal conflicts. The absence of a reliable model of care may make it more difficult to imagine oneself as a caregiver, thereby increasing vulnerability to psychological distress during this transitional period.

In contrast, women without histories of emotional abuse or neglect are more likely to develop a coherent sense of self, effective emotional regulation skills, and the capacity to provide sensitive and attuned care to their child. This is consistently supported by the literature, which emphasizes that the development of positive internal working models (MOI) is strongly promoted by supportive caregiving experiences (Bowlby, 1988; Mikulincer & Shaver, 2007). Such positive models are fundamental for fostering the quality of the mother–child relationship and for promoting secure attachment representations, which in turn serve as a foundation for healthy relational patterns and caregiving behaviors in adulthood.

Building on this theoretical framework and the evidence highlighting the lasting impact of early relational experiences on maternal functioning, our empirical findings further support these associations. Our univariate regression models revealed a significant association for all predictors examined. This would suggest that all the dimensions may be considered potential risk factors could indeed be related to the presence of antenatal depressive symptoms and psychological distress during pregnancy. In particular, the dimensions of emotional abuse and emotional neglect maintained a strong association with the presence of perinatal distress, in line with existing literature (Li et al., 2017). The finding that emotional abuse and emotional neglect are the most significant childhood adversities associated with perinatal outcomes highlights the critical role of emotional and relational components in the experience of pregnancy (Fu et al., 2024; Talmon et al., 2019). According to the literature, early experiences that compromise the parent–child attachment relationship may impair a mother’s ability to recognize and respond sensitively to her child’s need for care (Bowlby, 1982; Solomon & George, 2011). Women who experienced love and emotional support during childhood are generally more likely to offer similar care to their own children. In contrast, adverse childhood experiences such as emotional neglect can negatively affect their self-perception as capable and worthy parents (Kohlhoff & Barnett, 2013). Furthermore, individuals who have experienced childhood maltreatment often develop insecure internal working models, marked by mistrust and difficulty relying on others. In the context of parenting, these insecure models may lead to defensive and negatively skewed interpretations of a child’s behavior (Cassidy et al., 2013), thereby increasing the likelihood of adopting maladaptive parenting strategies (Bugental et al., 2002). From a clinical perspective, this view underscores the importance of early interventions aimed at improving emotional regulation, fostering a coherent sense of self, and consequently promoting more adaptive and functional behaviours during the transition to parenthood and in caregiving practices.

While the framework linking adverse childhood experiences to perinatal distress and later parenting difficulties is well supported, it may risk a somewhat deterministic interpretation, potentially underestimating resilience and the role of compensatory experiences or psychosocial support. Alternative explanations, such as genetic predispositions, socioeconomic stressors, and the buffering effects of partner or community support, may also contribute to the observed associations. A balanced perspective should therefore acknowledge variability in outcomes and the potential for positive change, avoiding the pathologization of individuals with adverse childhood histories.

According to the literature, which did not link physical abuse with the development of depressive risk before or after childbirth, our results confirm this aspect (Li et al., 2017; Robertson-Blackmore et al., 2013).

### 4.2. Social Support and Couple Relationship

In contrast to the existing literature, which consistently reports an inverse association between social support and antenatal depressive symptoms, where higher levels of perceived or received support are linked to lower levels of psychological distress during pregnancy (Dennis & Letourneau, 2007; Dennis & Ross, 2006), the present study did not identify a significant relationship between social support and antenatal depressive symptoms and psychological distress. One possible explanation for this discrepancy may lie in the characteristics of our sample, in which the majority of participants reported adequate to high levels of perceived social support. This limited variability could have reduced the likelihood of detecting statistically significant associations. Alternative explanations may derive from the use of an instrument that did not demonstrate adequate sensitivity within our specific sample, particularly considering our clinical experience, where relationships with the family of origin are commonly perceived as a pervasive and robust source of social support. One study (Laudani et al., 2014) indeed refers to the Mediterranean Model of Family Functioning, highlighting that families in Southern Europe tend to maintain stronger familial bonds, greater economic and psychological interdependence, and view the family as central to individual life. Furthermore, according to Santarelli and Cottone (2009), intergenerational support and proximity dynamics in Italy are distinctive, reflecting the tendency of young adult to leave the parental home at a relatively late age, which often results in sustained closeness and intensive support even after their marriage.

On the other hand, the dimension of relational well-being within the couple appears to be especially linked to psychological distress. Women who perceive their partner as emotionally supportive tend to report better psychological adjustment during pregnancy (Thomas et al., 2017); whereas those involved in an unsatisfactory or conflictual relationship are more likely to exhibit elevated levels of PND (Çankaya & Alan Dikmen, 2022). Furthermore, it is crucial to emphasize that the presence of depressive symptoms in one partner significantly increases the risk of the other partner developing similar difficulties (Thiel et al., 2020). Both maternal and paternal depression, though often expressed in different ways, can negatively affect parenting capacities and the ability to provide sensitive and responsive care to the child (Rollè et al., 2020). During the transition to parenthood the couple’s ability to mobilize their internal and relational resources plays a key role in promoting both parental adjustment and infant well-being. Our findings were consistent with this perspective, indicating that a satisfying couple relationship may function as a significant protective factor against the onset of perinatal psychological distress. In our sample, we identified approximately 5.3% of relational distress within the couples’ relationships.

### 4.3. The Partner Dimensions

It is important to acknowledge that the present study did not include data from partners, which represents a relevant limitation when interpreting our findings.

Given the reciprocal influence between maternal and paternal psychological well-being, and the evidence that depressive symptoms in one partner can exacerbate vulnerability in the other (Thiel et al., 2020; Rollè et al., 2020), excluding partners’ perspectives limits our ability to capture the bidirectional and dyadic nature of perinatal adjustment.

Including fathers’ perspectives would be particularly valuable, given that the transition to parenthood involves psychological, relational, and identity changes for men.

These include changes to roles, adjustments to intimate relationships, and an increase in financial and caregiving responsibilities.

These dynamics may in turn affect maternal health, paternal health, and the overall quality of the couple’s relationship. Future research, including both members of the couple, would therefore be essential to provide a more comprehensive understanding of the interplay between individual vulnerabilities, couple functioning, and perinatal psychological outcomes.

### 4.4. The Protective Role of Prenatal Attachment

Moreover, we found that the quality of prenatal attachment was a strong predictor of antenatal depressive symptoms. The emotional connection and the relationship quality with the unborn child are a considerable part of the child’s development in all aspects (Pisoni et al., 2014). Investigating this aspect, therefore, enables us to address various perspectives: in the realm of the mother’s psychological distress, as well as in the domain of the child’s well-being and that of the entire family.

Women who exhibit low levels of prenatal attachment combined with high levels of antenatal depressive symptoms tend to show low interest in the unborn child, focusing primarily on their own psychological distress and neglecting the development of a bond with the baby (Rollè et al., 2020). As noted by Seimyr et al. (2009), maternal awareness of the fetus, along with the feelings and perceptions directed toward the unborn child, contributes to increased maternal attentiveness to the child’s future well-being. Greater maternal awareness and emotional connection to the fetus facilitates the establishment of a secure attachment bond, which plays a crucial protective role in safeguarding both maternal mental health and the child’s emotional development from the risk of psychological distress (McNamara et al., 2019). In line with existing literature and supported by our findings, which suggest a potential protective role of prenatal attachment, it may be appropriate to consider the inclusion of this dimension into prenatal screening. Clinical screening tools for the assessment of prenatal attachment include standardized instruments such as the Maternal Antenatal Attachment Scale (MAAS) (Condon, 1993), which was employed in this study, as well as the Prenatal Attachment Inventory (PAI) (Muller, 1993). Integrating prenatal attachment assessment into clinical practice would facilitate the implementation of targeted psychoeducational interventions. These interventions aim to foster the development of a coherent maternal mental representation of the fetus, improve the mother’s understanding of fetal development, and encourage behaviors that enhance the early maternal–fetal bond throughout pregnancy. All these findings are cause for concern from a clinical perspective and suggest the need to look carefully at women’s well-being during pregnancy, starting with the attention that must be directed towards the various psychosocial risk factors that may contribute to its onset. Indeed, the most important element of screening these factors and managing high-risk situations lies precisely in its clinical relevance in terms of preventing distress and promoting physical, mental, and social well-being.

### 4.5. Clinical Implications

This work had highly significant clinical implications, as the data collection phase of patient screening directly informs clinical outcomes. Specifically, pregnant women who scored highly on the EPDS and PAMA scales, that evaluated perinatal depression (PND), were promptly referred for psychological support with a psychotherapist. This intervention was perceived as highly supportive by the patients, who appreciated the opportunity to receive continuous care during late pregnancy and the immediate postpartum period. The inclusion of psychologists within the obstetric and medical equipe enables targeted support for high-risk patients, as well as comprehensive care for all women both in the delivery room and postpartum. This approach facilitates a more holistic treatment of patients from both physical and psychological perspectives.

An important way to implement preventive and health-promoting interventions is through the provision of psychoeducational programs, such as prenatal education and birth preparation classes, which support expectant parents in developing coping skills and fostering emotional well-being during the transition to parenthood. In our project, these sessions are conducted within a multidisciplinary framework and involve the collaboration of various professionals, including psychologists, gynecologists, obstetricians, nutritionists, and neonatologists. Specifically, the program consists of nine sessions, four of which are led by psychologists. The first session addresses the psychological experiences of pregnancy; the second focuses on perinatal psychological distress and the significance of early attachment; the third explores couple dynamics, the changes associated with the transition to parenthood and in sexuality, as well as the redefinition of family roles; the fourth and final session discusses parental competences in the postpartum period, such as the management of crying and sleep. Each session includes experiential activities aimed at fostering participant reflection. The primary objective is to provide a supportive space for dialogue and guidance on key areas, including infant care, parenting roles, couples’ relationship quality, and the emotional and practical management of labor and childbirth.

Finally, by following up with patients approximately six months after childbirth, we can monitor their well-being and provide further psychological support if necessary. Future studies will provide additional results concerning the data collected six months postpartum with a particular focus on the role of partners.

### 4.6. Strengths and Limitations

This study presents preliminary findings collected during the first nine months following the launch of the project at the “Civico—Di Cristina—Benfratelli” Hospital in Palermo. As such, only data from the initial time point (T0), corresponding to the pregnancy period, have been included in the current analysis. The decision to report exclusively on T0 data at this stage was due to the ongoing nature of the study and the current sample size, which remains insufficient to support more advanced or longitudinal analyses. The data were collected through self-report questionnaires, administered under the careful supervision of clinical psychologists. While self-report measures may introduce certain biases, this method was considered the most appropriate to safeguard participants’ privacy and to reduce the intrusiveness of the assessment process. One important limitation of the present study lies in its cross-sectional design, which does not allow for causal inferences to be made. Although the results point to a potential causal association between the two dimensions examined, the lack of longitudinal data prevents us from establishing a direct cause-and-effect relationship.

It is also important to note that the present results refer exclusively to pregnant women. Although a screening protocol for partners was designed and initiated, the data collected to date are insufficient for inclusion in this initial analysis. The exclusion of partner data constitutes a limitation in relation to the project’s stated aim of investigating family and couple dynamics. Future analyses will incorporate partner data to provide a more comprehensive understanding of the dyadic and familial dimensions of perinatal mental health. Finally, potential selection bias should be acknowledged. Participation was voluntary and limited to a single hospital in southern Italy, which may have influenced the sociodemographic composition of the sample and limit the generalizability of the findings. Broader recruitment strategies and comparative analyses with national data will be pursued in the subsequent phases of the project.

## 5. Conclusions

In conclusion, this study, based on the project “When a Parent is Born,” presented preliminary results of data collection that is ongoing and which will be finalized approximately six months postpartum from a longitudinal perspective.

The preliminary evidence offered insight into the risk factors most closely associated with psychological distress during the perinatal period. By screening these factors during pregnancy, it is possible to consider interventions aimed at prevention and support. Recognizing women at high risk during pregnancy is essential and can contribute to ensuring optimal maternal and family health outcomes. In such cases, targeted clinical interventions, as outlined in this project, are implemented to provide comprehensive support and address the unique psychological and emotional experiences of these women. This proactive approach suggests potential benefits in mitigating the long-term consequences of postpartum depression and broader psychological distress, with an emphasis on promoting the well-being of the woman, her partner, the child, and the family as a whole.

According to the World Health Organization, caring for individuals, especially pregnant women and their children, necessitates attention not only to medical aspects but also to the overall well-being of the individual, including psychological, physical, and social factors. For this reason, it would be useful to have more concrete and practical guidelines, to be implemented through training programs aimed at health care personnel involved in perinatal care. This could facilitate the identification of any signs of distress, ensure more effective care of pregnant women, and improve overall management during this sensitive period.

This project aims to implement a collaborative effort among various professionals working as a team to provide optimal care to each patient, without overlooking how psychological factors can influence medical outcomes, and vice versa. This initiative represents a significant advancement within the Palermo area, and it is hoped that these findings may be generalizable to the broader Italian national context. The general aim is to establish a unified metric evaluation and develop a protocol for pregnancy management. Designed for implementation within a multidisciplinary framework, the protocol aims to promote overall maternal and infant well-being by integrating it into routine health service practices. Future research will generate comprehensive data across the various components of the project and further elucidate its clinical implications. Building upon the findings presented herein, subsequent studies will integrate the perspectives of partners and employ a longitudinal design, thereby enhancing the robustness of the current models and contributing additional nuanced insights.

## Figures and Tables

**Table 1:** Characteristics of the study population (Women N = 997).

Variable	Pregnant Women
**Age**	N (%)
<29 years old	231 (23.5%)
36–30 years old	532 (54.1%)
48–37 years old	220 (22.4%)
data missing	14 (1.4%)
**Nationality**	
Italy	983 (98.6%)
Other	14 (1.4%)
**Education**	
None	4 (0.4%)
Primary School certificate	8 (0.8%)
Middle school certificate	148 (14.8%)
High School diploma	361 (36.2%)
Bachelor’s degree	274 (27.5%)
Postgraduate specialization/Master’s degree	202 (20.3%)
**Employment status**	
Unemployed	78 (15.3%)
Housewife	215 (21.6%)
Student	19 (1.9%)
Precarious employment	98 (9.8%)
Stable employment	514 (51.6%)
Disability pension	1 (0.2%)
**Marital status**	
Single	96 (9.6%)
Married/Cohabitant	893 (89.6%)
Separated/Divorced	8 (0.8%)
**Economic status**	
Severe problems (debts, unable to pay rent, etc.)	4 (0.4%)
Some problems (limitation of daily expenses, cannot afford vacations)	85 (8.5%)
Modest standard, but without particular difficulties	612 (61.4%)
Medium–high (own a house, frequent vacations, etc.)	296 (29.7%)
**Gestational age**	
First trimester	14 (1.4%)
Second trimester	76 (7.6%)
Third trimester	907 (91%)
**Pregnancy**	
Planned pregnancy	706 (70.8%)
Unplanned pregnancy	291 (29.2%)
**First pregnancy**	
Yes	611 (61.3%)
No	386 (38.7%)
**Other children**	
Yes	386 (38.7%)
No	611 (61.3%)
**High-risk pregnancy**	
Yes	273 (27.4%)
No	724 (72.6%)
**Medically Assisted Procreation**	
Yes	58 (5.8%)
No	938 (94.1%)
**Psychopharmacotherapy**	
Yes	13 (1.3%)
No	984 (98.7%)
**Previous high-risk pregnancies**	
Yes	49 (4.9%)
No	948 (95.1%)
**Miscarriage**	
Yes	227 (22.8%)
No	770 (77.2%)
**Voluntary termination of pregnancy**	
Yes	55 (5.5%)
No	942 (94.5%)
**IUFD Intrauterine fetal death**	
Yes	5 (0.5%)
No	992 (99.5%)
**Prenatal class**	
Yes	465 (46.6%)
No	532 (53.4%)
**Type of pregnancy**	
Single pregnancy	987 (99%)
Twin pregnancy	10 (1.0%)

**Table 2:** Summary of instruments.

Instrument	Purpose	Dimensions	Cut-Off/Interpretation
Edinburgh Postnatal Depression Scale (EPDS)	Assesses emotional distress over the past week	Single scale (10 items); item 10 screens for suicidal ideation	0–8: No risk; 9–11: Medium risk; 12–30: High risk
Perinatal Assessment of Maternal Affectivity (PAMA)	Screens for perinatal affective disorders	Emotional and behavioral difficulties (e.g., anxiety, depression, stress, addictions)	No standardized cut-off available; higher scores indicate greater risk
Maternity Social Support Scale (MSSS)	Assesses perceived social support	Single scale (support from family, partner, friends)	6–18: Low; 19–24: Intermediate; >24: Adequate support
Dyadic Adjustment Scale (DAS)	Measures romantic relationship satisfaction	Dyadic Satisfaction, Dyadic Cohesion (only these used)	<21: Relational distress
Childhood Trauma Questionnaire—Short Form (CTQ–SF)	Assesses childhood abuse and neglect	5 subscales: Emotional Abuse, Physical Abuse, Sexual Abuse, Emotional Neglect, Physical Neglect	EA ≥ 13; PA ≥ 10; SA ≥ 8; EN ≥ 15; PN ≥ 10 (moderate–severe trauma)
Maternal Antenatal Attachment Scale (MAAS)	Evaluates prenatal attachment	Quality of attachment; Intensity of concern	Higher scores = Higher levels of attachment

**Table 3:** Descriptive assessment instruments.

Variable	Pregnant Women
**MSSS**	N (%)
Low	76 (7.6%)
Moderate	327 (32.8%)
High	594 (59.6%)
**CTQ**	
Emotional Abuse	20 (2.0%)
Physical Abuse	21 (2.1%)
Sexual Abuse	14 (1.4%)
Emotional Neglect	30 (3%)
Physical Neglect	11 (1.1%)
**MAAS**	
Low (<72)	42 (4.2%)
Moderate (72–84)	555 (55.7%)
High (>84)	400 (40.1%)
**EPDS**	
None/low	750 (75.2%)
Moderate	113 (11.3%)
High	134 (13.4%)
**EPDS**	
Yes	24 (2.4%)
No	973 (97.6%)
**PAMA**	
Anxiety	386 (38.7%)
Depression	89 (8.9%)
Stress	215 (21.5%)
Irritability	183 (18.3%)
Relational Problems	85 (8.5%)
Psychosomatic Problems	369 (37%)
Physiological Problems	370 (37.1%)
Addictions	33 (3.3%)
**DAS**	
Relational well-being	805 (93.2%)
Relational distress	45 (5.3%)
Data missing	147 (14.7%)

**Table 4:** Univariate and multivariate linear regression.

Independent Variables	Univariate Analyses			Multivariate Analyses (CTQ Subscales)		
Association with EPDS	β	95% CI	*p*-value	β	95% CI	*p*-value
MSSS Total score	−0.110	−0.20; −0.05	0.001 **			
MAAS Total score	−0.234	−0.25; −0.14	<0.001 ***			
Emotional abuse	0.205	0.28; 0.51	<0.001 ***	0.106	0.05; 0.35	0.006
Physical abuse	0.106	0.11; 0.44	0.001 **	−0.032	−0.30; 0.13	0.441
Sexual abuse	0.108	0.14; 0.51	0.001 **	0.057	−0.06; 0.41	0.152
Emotional neglect	0.253	0.27; 0.44	<0.001 ***	0.209	0.18; 0.40	<0.001 ***
Physical neglect	0.140	0.24; 0.62	<0.001 ***	−0.014	−0.27; 0.18	0.709
DAS Total score	−0.278	−0.28; −0.17	<0.001 ***			
Association with PAMA						
MSSS Total score	−0.090	−0.17; −0.03	0.004 *			
MAAS Total score	−0.184	−0.19; −0.09	<0.001 ***			
Emotional abuse	0.252	0.35; 0.57	<0.001 ***	0.165	0.16; 0.44	<0.001 ***
Physical abuse	0.147	0.21; 0.52	<0.001 ***	0.014	−0.16; 0.23	0.736
Sexual abuse	0.115	0.15; 0.51	<0.001 ***	0.033	−0.13; 0.32	0.413
Emotional neglect	0.235	0.23; 0.39	<0.001 ***	0.171	0.12; 0.33	<0.001 ***
Physical neglect	0.113	0.14; 0.51	<0.001 ***	−0.043	−0.34; 0.09	0.261
DAS Total score	−0.262	−0.25; −0.15	<0.001 ***			

* *p* < 0.05; ** *p* < 0.01; *** *p* < 0.001.

**Table 5:** Multivariate linear regression (subsample: N = 850).

Indipendent Variables	Multivariate Analyses (N = 850)		
Association with EPDS	β	95% CI	*p*-value
MSSS Total score	−0.048	−0.141; 0.020	0.143
MAAS Total score	−0.120	−0.164; −0.047	<0.001 ***
Emotional abuse	0.125	0.072; 0.429	0.006
Physical abuse	−0.074	−0.483; 0.025	0.077
Sexual abuse	0.048	−0.107; 0.469	0.218
Emotional neglect	0.125	0.056; 0.310	0.005 *
Physical neglect	−0.022	−0.321; 0.176	0.565
DAS Total score	−0.226	−0.305; −0.165	<0.001 ***
Association with PAMA			
MSSS Total score	−0.032	−0.113; 0.038	0.328
MAAS Total score	−0.081	−0.122; −0.012	0.017
Emotional abuse	0.222	0.249; 0.584	<0.001 ***
Physical abuse	−0.024	−0.308; 0.168	0.564
Sexual abuse	0.019	−0.205; 0.336	0.634
Emotional neglect	0.061	−0.035; 0.204	0.167
Physical neglect	−0.068	−0.439; 0.027	0.083
DAS Total score	−0.233	−0.293; −0.161	<0.001 ***

* *p* < 0.05; *** *p* < 0.001.

## Data Availability

The datasets used and analyzed during the current study are available from the corresponding author on reasonable request.

## Abbreviations

The following abbreviations are used in this manuscript:

PND	Perinatal Depression
PPD	Postpartum Depression
